# The PreserFlo MicroShunt in the Context of Minimally Invasive Glaucoma Surgery: A Narrative Review

**DOI:** 10.3390/ijerph20042904

**Published:** 2023-02-07

**Authors:** Emil Saeed, Kinga Gołaszewska, Diana Anna Dmuchowska, Renata Zalewska, Joanna Konopińska

**Affiliations:** Department of Ophthalmology, Medical University of Bialystok, Kilinskiego 1 STR, 15-089 Bialystok, Poland

**Keywords:** aqueous humor, cytostatic agents, open-angle glaucoma, reoperation, surgical techniques, trabeculectomy

## Abstract

Recently, the quest for novel glaucoma surgical techniques and devices has been underway. Trabeculectomy remains the gold standard, but it requires the implantation of glaucoma drainage devices and frequent follow-ups, and it also carries a high risk of serious complications. The need for less invasive and safer procedures has led to the development of minimally invasive glaucoma surgery (MIGS), particularly for patients with mild–to–moderate disease. Among them, minimally invasive bleb surgery seems to be effective in classical glaucoma surgery, while maintaining MIGS benefits. The relatively new PreserFlo^®^ MicroShunt (Santen, Osaka, Japan) is registered in Europe. It was released in 2019 for the treatment of patients with early–to–advanced open-angle glaucoma, where intraocular pressure (IOP) remains uncontrolled while on maximum tolerated medication and/or where glaucoma progression warrants surgery. This review focuses on the place of the PreserFlo MicroShunt, characterized by ab externo implantation, among MIGS procedures, discussing its advantages and disadvantages. The mechanisms of action, technical aspects, efficacy, and safety issues are summarized. The surgical technique, its efficacy, and safety profile are described, and directions for future studies are indicated. The PreserFlo MicroShunt ensures a high safety profile, minimal anatomical disruption, meaningful IOP-lowering effect, and ease of use for patients and physicians.

## 1. Introduction

Trabeculectomy has been the gold standard for glaucoma surgery for over 50 years [1]. However, it may cause many short- and long-term side effects, and its effectiveness decreases over time [2,3]. For over a decade, intensive research has been conducted on safer surgical techniques, such as minimally invasive glaucoma surgery methods, which have been termed microinvasive glaucoma surgery (MIGS). Over the last few years, these devices have been developed and gained growing interest [4,5]. In 2012, Saheb and Ahmed characterized the five properties of MIGS [6]. These include an ab interno approach through a clear corneal incision, which spares the conjunctiva; a minimally traumatic procedure on the target tissue; an intraocular pressure (IOP)-lowering efficacy that justifies the approach; a high safety profile avoiding serious complications compared to other glaucoma surgeries; and a rapid recovery with minimal impact on the patient’s quality of life. In 2014, the American Glaucoma Society and the US Food and Drug Administration (FDA) recognized that MIGS is characterized by the implantation of a surgical device intended to lower IOP via an outflow mechanism with either an ab interno or ab externo approach, associated with very little or no scleral dissection [7,8]. None of the devices require cutting off the sclera, and most are placed in the anterior chamber (AC) with a clear corneal incision. Therefore, they are often used in combination with phacoemulsification and implantation of an intraocular lens [9,10]. Recently, Qidawi et al. [11] introduced the term minimally invasive bleb surgery (MIBS) to differentiate the latter group from traditional MIGS. It combines the properties of traditional glaucoma surgery with those of MIGS. Thanks to microstenting, MIBS standardizes bleb surgeries. Despite the similarity between these two groups, they are intended for two different populations of patients with glaucoma. MIGS is suitable for patients with mild–to–moderate glaucoma who fail to adhere to pharmacological treatment and are at risk of glaucoma progression if left untreated. MIBS is designed for patients with moderate–to–severe glaucoma and a high risk of progression, despite pharmacological treatment. The advantages of this technique include a strong IOP-lowering effect, with standardized lumen size, minimal risk of hypotony, greater comfort due to posterior bleb formation (at the same time, lower risk of inflammation), and reduced postoperative care intensity. On the other hand, it still requires the use of cytostatic agents (mitomycin C [MMC] or 5-fluorouracil [5-FU]) to address wound healing. There are already articles discussing the problem [12], but in this paper, the authors describe and compare PreserFlo with numerous MIGS procedures.

## 2. MIGS and MIBS

In the quest for the perfect glaucoma surgery in the last decade, many innovations and devices have appeared on the market. Depending on the mechanism of the IOP-lowering action, MIGS implants can be classified as those that improve conventional outflow, enhance unconventional outflow, or bypass conventional outflow. Based on the filtering bleb dependency, these implants can be classified as bleb-dependent or bleb-independent [5,13] (Table 1).
Bleb-independent MIGS: Schlemm’s canal-based procedures:   Implants: iStent^®^, iStent inject^®^, and Hydrus^®^ microstent [13,14,15,16,17]  Incision surgery: goniotomy with Kahook Dual Blade, gonioscopy-assisted    transluminal trabeculotomy, and trabectome [18,19]   Viscodilation: ab interno canaloplasty and goniotomy with the OMNI surgical system Lowered aqueous humor production:  Endoscopic cylclophotocoagulation [20,21,22,23,24,25] Suprachoroidal space implants:  Implants strengthen the outflow via an unconventional route: CyPass^®^, iStent    Supra^®^, and MINIject [26,27,28] Bleb-dependent MIGS, i.e., MIBS:  Implants bypass physiological aqueous drainage pathways and are conjuncti-va-   dependent: XEN^®^ Gel Stent and PreserFlo MicroShunt [29]

## 3. The PreserFlo MicroShunt

The PreserFlo MicroShunt (Santen, Osaka, Japan), formerly known as the InnFocus MicroShunt, is implanted using the ab externo approach, and it fulfills the MIGS criteria proposed by the American Glaucoma Society and the US FDA [7]. The MicroShunt is registered in Europe and was released in 2019 for the surgical treatment of patients with early–to–advanced primary open-angle glaucoma (POAG), where the IOP remains uncontrolled while on maximum tolerated medical therapy and/or where glaucoma progression warrants surgery. It received its CE mark in 2012 [30,31]. It has not yet been approved by the US FDA.

### 3.1. Mechanism of Action

The PreserFlo MicroShunt uses the same surgical pathway as trabeculectomy [31]. Similar to classical surgery, it drains the aqueous humor from the AC to a fornix-based subconjunctival/Tenon’s flap [32]. It bypasses the trabecular meshwork, which is assumed to be the place with the highest resistance for drainage of aqueous humor [5,33]. Drainage from the bleb then follows the path of least resistance into the episcleral venous system and/or occurs through microcysts in the conjunctiva into the tear film [32]. Compared to trabeculectomy, the bleb is placed more posteriorly and thicker than in the conventional filtering procedure [34].

The design of the PreserFlo MicroShunt device is based on the Hagen–Poiseuille equation [35], similar to that of the Xen Gel Stent. The diameter of the tube is 350 µm; therefore, the device requires only a minimally invasive surgical incision tract for entry into the AC. The overall length of the device (8.5 mm) and lumen size (70 µm) are carefully balanced to avoid blockage, prevent hypotony, and provide a controlled reduction in IOP [35]. Partway down the tube, a set of wings or fins provides a means to hold the device firmly in place in the sclera, prevent leakage around the tube, and maintain the correct orientation of the bevel within the AC. The MicroShunt does not include a plate, which makes the surgical procedure less traumatic than a device with a plate (Ahmed (New World Medical^®^, Rancho Cucamonga, CA, USA) and Molteno (Nova Eye Medical, Fremont, CA, USA) devices).

It is made from a biostable, biocompatible material called poly(styrene-block-isobutylene-block-styrene) (SIBS). This material has several important advantages when used in glaucoma surgery [32,36,37]. SIBS is soft and flexible, meaning that it easily conforms to the curvature of the eye and reduces the risk of the device causing erosion of the sclera. SIBS has no degradable bonds and virtually no foreign-body reaction in vivo, which manifests in the eye as clinically insignificant inflammation, with no scarring or capsule formation [37]. The clinical use of SIBS has been demonstrated in other therapeutic areas [36,37,38]. It has been used safely as a coating for the TAXUS^®^ Express™ cardiac stent, which has been investigated in various clinical trials spanning 15 years and has been implanted in millions of patients. Finally, the non-animal source of SIBS provides an option for patients and clinicians who wish to avoid using animal-based materials (Xen Gel implant) [31,39].

### 3.2. Preparation for the Surgical Procedure

The implantation of a PreserFlo MicroShunt takes about 12 min and does not require the creation of a scleral flap or the use of sclerostomy, iridotomy, or tensioning sutures [40]. This makes the implantation procedure much less invasive than trabeculectomy, while still using the trabeculectomy pathway, which is very effective in lowering IOP. There is no additional complexity in the procedure compared to trabeculectomy, and no specialized equipment is required [41].

The recovery time after PreserFlo MicroShunt implantation is generally shorter, and the follow-up visits are fewer than those after a classical procedure. The recovery time is typically approximately 2 weeks for a PreserFlo MicroShunt, whereas the recovery time from trabeculectomy is generally between 4 and 6 weeks.

Postoperative interventions, such as suture lysis or goniopuncture, are not required [31,39]. For the first series of patients on whom the procedure is performed, it is recommended that the PreserFlo implantation is not combined with cataract extraction because of the potential for an increased level of inflammation in the AC. For glaucoma surgeons already experienced with the procedure, it is possible to combine the procedure with cataract surgery if desired [39].

To prevent intraoperative complications, patients with narrow palpebral fissures, deep-set eyes, narrow AC angles less than Shaffer Grade 3, significant arcus senilis, or dilated pupils should preferably not be selected for this procedure [41].

The PreserFlo MicroShunt device is supplied with a comprehensive set that includes most of the materials required for its implantation: the MicroShunt, a 3 mm scleral marker, a 1 mm slit-angled knife, a 25-gauge needle, a 23-gauge thin-wall cannula, and a marker pen. Additional equipment and materials that may be needed include three laser in situ keratomileusis (LASIK) shield sponges, conjunctival forceps, Bonn forceps, blunt dissection scissors, spring/conjunctival scissors, spears, bipolar diathermy equipment, suture material, syringes, and balanced salt solution.

Before the PreserFlo MicroShunt implantation procedure, the glaucoma surgeon must first determine the desired position for device placement. If possible, similar to trabeculectomy, it should be placed in a superior position fully protected by the upper eyelid, either nasally or temporally, to avoid the extraocular muscles. Moreover, interpalpebral or inferior fornix filtering blebs have an up to 10 times greater incidence of inflammation and endophthalmitis. However, if previous surgery had been performed in either of these quadrants, an inferior quadrant can be considered. Recurrent subconjunctival hemorrhage has been reported more commonly with inferior filtering blebs, together with bleb discomfort and pain [41].

### 3.3. Surgical Procedure

The first step in the PreserFlo MicroShunt implantation procedure is to anesthetize the eye based on the surgeon’s preference. If the surgeon considers it necessary, a traction suture can be used to keep the position of the eye fixed.

Next, the conjunctiva is opened in the limbus. A deep, wide pocket is created by dissecting the Tenon’s capsule from the sclera using blunt scissors. The pocket should be created using the Peng–Khaw technique, with a 6–8 mm incision at the corneoscleral junction to form a fornix-based sub-Tenon’s/sub-conjunctival flap with a width of 90–120° and a depth of at least 8 mm. The marker included in the PreserFlo MicroShunt kit can be used to gauge the depth of the pocket.

Bleeding is controlled using bipolar diathermy. This is preferred to cauterization, as it minimizes the amount of thermal damage exerted and any undesirable healing processes in the adjacent tissue. Diathermy should be performed, according to standard practices and the preferences of the glaucoma surgeon [42].

At the surgeon’s discretion, the application of three 0.2–0.4 mg/mL MMC-soaked corneal LASIK shield sponges under the subconjunctival pouch over a circumference of 90–120° approximately 8 mm deep for 2–3 min can be considered. The conjunctival wound edge should not come into contact with the antimetabolite. The sponges are removed, and the flap is rinsed with generous amounts of balanced saline solution (at least 20 mL).

Next, the incision site is marked. Using the marker supplied in the PreserFlo MicroShunt kit, a mark is placed on the sclera 3 mm from the middle border of the surgical limbus to the sclera.

After cauterization of the flap area, the 1 mm slit-angled knife supplied in the PreserFlo MicroShunt kit is used to create a 2 mm shallow tunnel in the sclera.

Then, the 25-gauge needle supplied in the PreserFlo MicroShunt kit is passed through the pocket created in the previous step and used to create a needle tract that opens into the AC. To create the needle tract successfully, the needle should be introduced parallel to the sclera, and the angle of the needle should be changed prior to reaching the middle border of the surgical limbus to enter the AC at the level of the trabecular meshwork, bisecting the angle between the iris and cornea. Alternatively, the angle of the needle can be kept constant, and the eye is rotated toward the surgeon to provide the same effect.

**Figure 1:**
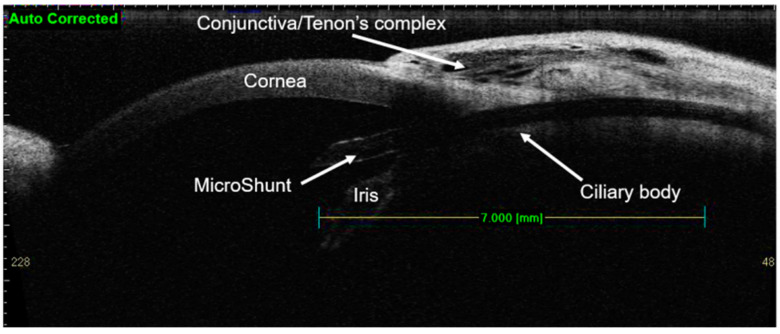
PreserFlo MicroShunt implantation. Source: https://eyewiki.aao.org/PreserFlo_Ab-Externo_MicroShunt (accessed on 30 November 2022).

Next, the PreserFlo MicroShunt is inserted into the scleral tunnel. The device is threaded through the needle tract, bevel upward, until the proximal end is seen within the AC, and the wedge fins are locked into the scleral pocket. The proximal tip of the shunt is then checked to observe the AH flow. If no flow is observed, the 23-gauge thin-wall cannula should be used to flush the distal end of the device with a balanced salt solution to initiate flow. Gentle pressure can be applied to the cornea. If a flow is still not observed, the surgeon may consider filling the AC through paracentesis with a balanced salt solution or flushing the tip of the MicroShunt from the distal end with the 23-gauge thin-wall cannula. Another possibility is the repositioning of the tube in a newly created knife-and-needle tract (Figure 1).

Once AH flow is confirmed, the conjunctiva is closed. The PreserFlo MicroShunt is tucked under the Tenon’s capsule, taking care to position it straight and flat on the sclera. The Tenon’s capsule is then pulled up and anchored with a suture, and the conjunctiva is closed in a watertight fashion, according to the surgeon’s choice of suture material.

### 3.4. Efficacy of the PreserFlo MicroShunt

The literature in this field is still limited. The results of existing studies indicate a mean IOP reduction of 30–55% from baseline and a substantial reduction in medication burden [43]. The longest retrospective observational study to date reports 5-year outcomes in 23 mixed-race patients with moderate–to–severe POAG in the Dominican Republic, who underwent PreserFlo MicroShunt implantation. This single-center, nonrandomized trial was completed in 2016 [44]. Patients were 18–85 years old, had uncontrolled glaucoma with maximum tolerated doses of medications (IOP 18–40 mmHg), and showed glaucomatous neuropathy progression manifesting as visual field deterioration or optic disc changes. The exclusion criteria were a vision level of no light perception; a known allergy to MMC; a history of previous ophthalmic surgery, except for uncomplicated phacoemulsification cataract surgery; corneal refractive surgery; or a need for glaucoma surgery combined with other ocular procedures, except for cataract surgery [39,44]. The primary efficacy endpoints were a reduction in medicated IOP relative to the preoperative value and the success rate, defined as the percentage of patients without pressure failures or surgical failures. Pressure failures were defined as an IOP beyond the target range (6–21 or 6–14 mmHg) or with <20% reduction from baseline on two consecutive follow-up visits after 3 months and <20% reduction below baseline at the last visit, at which the success rate was reported. Surgical failure was defined as patients requiring reoperation in the operating room, excluding bleb needling. The follow-up visits were conducted on day 1, day 7, week 3, week 6, month 3, month 6, and annually following month 12. The primary safety endpoint was the incidence of all device- or procedure-related adverse events during the study. Success was reported as overall (with or without concomitant use of glaucoma medications), qualified (with glaucoma medications), or complete (without glaucoma medications). The reduction in IOP observed at year 3 was sustained over years 4 and 5. Mean IOP ± standard deviation was reduced from 23.8 ± 5.3 mmHg to 12.8 ± 5.6 mmHg at year 4 (*n* = 21) and 12.4 ± 6.5 mmHg at year 5 (*n* = 21), representing a mean decrease from baseline of 45.7% and 46.7%, respectively. At years 4 (*n* = 20) and 5 (*n* = 19), >80% of patients achieved overall success, with an IOP of 6–21 mmHg. Approximately 70% (*n* = 16) and 57% (*n* = 13) of patients achieved complete success at years 4 and 5, respectively. Moreover, the mean number of glaucoma medications received by patients in the same study was substantially reduced after five years of follow-up. At baseline, the mean number of glaucoma medications administered per patient was 2.4. At years 1 and 5, this was reduced to 0.3 and 0.8, respectively. At year 5, this constituted a mean reduction of 66% in the number of medications compared to baseline. A total of 61% of patients were taking no glaucoma medications at all at year 5.

A recent publication by Beckers et al. [45] reported a prospective, multicenter, open-label, single-arm study performed in France, the Netherlands, Spain, and Switzerland that examined the safety and efficacy of the PreserFlo MicroShunt over 2 years. MMC was used as an adjunct to PreserFlo MicroShunt implantation, and the concentration and exposure time varied among sites. The doses were 0.2 mg/mL and 0.4 mg/mL, and the exposure time was either 2 or 3 min. Patients were aged between 18 and 85 years and had POAG that was not controlled with maximum tolerated medical therapy (with medicated IOP ≥ 18 and ≤35 mmHg). Exclusion criteria were previous incisional ophthalmic surgery other than uncomplicated cataract surgery at least 6 months before study enrolment. Patients were also excluded if they had no light perception, required glaucoma surgery combined with other ocular procedures, or had an anticipated need for additional ocular surgery in the study eye during the study period. The primary efficacy endpoint was IOP reduction after 1 year of follow-up, with the determination of success at year 1 (defined as patients who were not considered to have pressure or surgical failures). Pressure failures were defined as patients out of the target IOP range (between ≥6 and <21, <18, or <15 mmHg) or with <20% reduction from baseline on 2 consecutive scheduled follow-up visits every 3 months and <20% reduction below baseline at the last visit, at which time the success rate was reported. Surgical failure was defined as patients requiring additional glaucoma surgery in the operating room (e.g., bleb revision, new device implantation, trabeculectomy, or repositioning of the device). The primary safety endpoint was the incidence of all device- or procedure-related complications in the study eye. Follow-up visits were carried out preoperatively, and further tests were conducted postoperatively on day 1, day 7, week 4, month 3, month 6, month 9, year 1, and year 2. A total of 81 patients were enrolled in this study. At baseline, the population had a mean age of 64 years, 36% were male, and the majority (55%) were phakic. The mean medicated IOP was 21.7 mmHg, with 36% of patients having an IOP >21 mmHg. Patients in the study group were taking a mean of 2.0 glaucoma medications. At year 2, the mean IOP was 14.1 mmHg, a reduction of 35% from the mean preoperative IOP of 21.7 mmHg. In addition, 74.1% of patients achieved treatment success, defined as the absence of 2 consecutive pressure failures, i.e., an IOP outside the target range or <20% reduction from baseline, with and without supplemental glaucoma medication use. Overall success was defined as the absence of 2 consecutive pressure failures, i.e., an IOP beyond the target range or <20% reduction from baseline, with or without supplemental glaucoma medication use. Complete success was reported when patients did not require supplemental glaucoma medication to maintain controlled IOP levels. Qualified success was reported when patients required supplemental glaucoma medication to maintain controlled IOP levels.

The IOP reductions after PreserFlo MicroShunt implantation at years 1 and 2 were independent of the MMC dose used during the procedure. In a post hoc analysis of subgroups according to MMC dose, both groups had similar IOP reductions from baseline at year 2. The success rates at year 2 were also similar between the two MMC dose groups. Compared to the MMC 0.2 mg/mL subgroup, significantly more patients in the MMC 0.4 mg/mL subgroup were medication-free at year 2 (*p* < 0.05). From month 6 onward, significant differences in medication reduction were found between the groups: 90.3% of patients in the 0.4 mg/mL MMC group were medication-free at year 2, compared to 50.1% of the patients in the 0.2 mg/mL MMC group. The significantly higher medication use from month 6 onward in patients with 0.2 mg/mL MMC may have been caused by bleb fibrosis reducing the aqueous outflow in these patients. Alternatively, different prescribing practices for antiglaucoma eye drops among sites may have contributed to the higher use of medications in patients receiving 0.2 mg/mL MMC.

Fea et al. [46] reported the IOP-lowering effect of the PreserFlo MicroShunt in 104 eyes of 104 patients with POAG or pseudoexfoliative glaucoma (PXG) in a retrospective, multicenter study (4 sites in Italy, 1 in Sweden, and 1 in the United Kingdom) from 2021 [25]. Patients included in the study (age 71.4 ± 12.6 years and 45 [43.3%] women) had poor treatment adherence or intolerance to glaucoma drops. Patients with phacodonesis, pemphigoid, or conjunctival scarring were excluded from analysis. Patients with any form of glaucoma other than POAG/PXG or with cataracts requiring surgical intervention were excluded from the study. The standard surgical procedure was performed. Topical MMC (0.2 to 0.5 mg/mL) was placed into the subconjunctival space using 3 LASIK shields for 2–3 min (MMC concentration and exposure time were decided based on the surgeon’s preference and the patient’s characteristics). The follow-up visits were on days 1 and 7 (±1 day) and months 1, 3, 6, 9, and 12 (±15 days). Complete success was defined as an IOP ≤18 mmHg and an IOP reduction ≥20% without any hypotensive medication after 12 months and qualified success as an IOP ≤18 mmHg and an IOP reduction ≥20% with glaucoma drops. Failure was defined as an IOP <4 mmHg on more than 2 consecutive visits, or if patients needed further glaucoma surgery. The primary endpoints were the mean change in IOP and the number of glaucoma medications from baseline to month 12. Secondary endpoints included the proportion of patients achieving a month-12 IOP ≤ 21 mmHg, IOP ≤ 18 mmHg, IOP ≤ 16 mmHg, and ≤14 mmHg, irrespective of the percentage reduction. Mean IOP lowering was −16.6 (−14.9 to −18.3) mmHg; −14.8 (−12.9 to −16.7) mmHg; −11.32 (−9.3 to −13.3) mmHg; −11.2 (−9.4 to −13.0) mmHg; −10.2 (−8.5 to −11.9) mmHg; −10.5 (−9.0 to −13.0) mmHg; and −10.1 (−8.5 to −11.8) mmHg at day 1, week 1, and months 1, 3, 6, 9, and 12, respectively. In total, 26.0% of eyes were categorized as a complete success and 58.7% as a qualified success. Eyes with POAG (mean difference −10.7 ± 5.5) had greater IOP reduction compared to those with PXG (−11.5 ± 4.5), which was not a significant difference between the two glaucoma types. At month 12, glaucoma medications were significantly reduced from 3.0 ± 1.0 to 0.8 ± 1.0 *p* = 0.01. Regarding the reduction in glaucoma medications, differences between eyes with POAG and those with PXG and between phakic and pseudophakic eyes were not significant. The most common complications were hyphema (8 eyes, 7.7%) and choroidal detachment (5 eyes, 4.8%).

### 3.5. Safety

Adverse events occur in 10–25% of cases and include hyphema (<10%), hypotony (10–16%), a shallow AC (4–13%), choroidal detachment or effusion (<9%), the device touching the iris (13%), and exposure of the Tenon’s capsule (9%) [45]. As it is a bleb-dependent procedure, it might require bleb needling in 2–10% of cases, usually within 9 months of follow-up (34, 45). However, it may substantially reduce IOP, in contrast to most MIGS procedures, which are associated with only modest reductions in IOP; therefore, it can target patients with moderate–to–severe and refractory glaucoma. Only a few clinical trials with the PreserFlo MicroShunt are currently underway (NCT01881425, NCT00772330, NCT01563237, and NCT02177123; https://www.clinicaltrials.gov/ (accessed on 15 September 2022).

In the study by Fea et al. [46], all complications were mild and successfully resolved. Needling was performed in 19 (18.3%) eyes and surgical revision in 14 (13.5%) eyes. In total, 4 (3.9%) eyes underwent both procedures. It is striking that there were no significant differences in age between patients who did not undergo needling or surgical revision and those who did. In total, 9 (8.7%) eyes received digital ocular massage, and 5 (4.8%) and 3 (2.9%) eyes underwent postoperative subconjunctival injections with MMC and 5-FU, respectively. In total, 9 patients experienced a reduction in best corrected visual acuity of >2 lines, 8 due to cataract progression and 1 due to macular edema [26].

In the study by Beckers et al., non-serious adverse events occurred in 57 (56.4%) patients (increased IOP, hypotony, keratitis, bleb leakage, flat AC, hyphema, and diplopia), whereas serious adverse events occurred in 7 (6.9%) patients (keratitis, conjunctival dehiscence, and corneal ulcer) [45].

In the study by Batlle et al. [44], a total of 21 non-serious complications were reported during the first 3 years after surgery; an additional 10 non-serious adverse events and 4 serious adverse events (posterior capsule opacification [*n* = 2, 8.7%], posterior synechiae [*n* = 1, 4.3%], and pupillary capture [*n* = 1, 4.3%]) were reported by year 5. At year 5, there were no reports of device degradation, chronic hypotony, or endophthalmitis. Reoperation was performed in 2 patients (8.7%) because of bleb failure. A second MicroShunt was implanted in one of these patients, whereas the MicroShunt was replaced by a XEN 45 Gel Stent in the second patient. Bleb needling was required in 2 patients (8.7%) [44].

Scheres et al. showed that MicroShunt Preserflo comparing to XEN Gel Stent achieved comparable results in POAG eyes with a similar high safety profile with at least 6 months of follow-up. In total, 73% of eyes with Xen and 79% of Preserflo implantations showed qualified success after 24 months of follow-up. The mean number of medications was also reduced in both groups, with more than half of the patients without the need for ongoing topical medications after 24 months [47].

The efficacy of the PreserFlo depends on the placement and concentration of MMC, as shown in the study by Riss et al. [48]. Patients treated with 0.4 mg/mL MMC placed close to the limbus presented a 55% reduction in IOP, whereas eyes with the same concentration of MMC placed deep in the pocket presented a 32% reduction in IOP. The alkylating and antifibrotic properties of MMC that exert toxic effects on corneal endothelial cells, keratocytes, and limbal stem cells must be taken into account [27].

According to a study conducted by Qidwai et al. [11], the PreserFlo MicroShunt was associated with fewer postoperative bleb manipulations than the XEN 45 in a 24-month observation. In the PreserFlo group, 12 (27.2%) patients required 5-FU injections after 3 months. Among MicroShunt patients, 4 (8.3%) bleb needlings and 4 (8.3%) bleb revisions were required. In the XEN 45 group, 10 (27%) patients underwent 5-FU injections, and 12 (32%) required bleb needling. The bleb revision rate was comparable to that of the PreserFlo. One (2.7%) XEN was replaced with a PreserFlo MicroShunt. One patient in the PreserFlo group developed suture-related keratitis, which was successfully treated but led to cystoid macular edema, which resolved without any persistent effect. In patients with PreserFlo implantation, the average central endothelial cell count was 1963.2  ±  429.0 at baseline and remained mostly stable at 1930.1  ±  538.0 (*p*  =  0.98) until the end of the observation period, but 2-year follow-up data for central endothelial cell counts were only available for 14 patients (29.2%) [28].

These results are in the line with the three-year study conducted by Pawiroredjo et al. [49], which showed that the mean reduction in preoperative IOP and glaucoma medications for the standalone and combined procedures were similar: 5.07 mmHg (23.6%) and 8.92 mmHg (38.2%; *p* = 0.234), and 2.67 (75.5%) and 1.62 (56.8%; *p* = 0.830), respectively, at the last postoperative visit. The most common complications in this study were hypotony (1.7–39%) and choroidal effusion (2.0–12.9%).

## 4. Discussion

Currently, the literature on the PreserFlo MicroShunt is limited to short-term follow-up, small case numbers, and mostly observational studies, but these initial studies have shown encouraging results on the IOP-lowering efficacy and safety of this device [11,41,44,45,50]. To date, there is only one randomized control trial comparing trabeculectomy with PreserFlo MicroShunt implantation [51]. The 1-year interim results showed a mean IOP reduction from 21.1 mmHg to 14.3 mmHg, with a mean of 0.6 postoperative glaucoma medications, in the PreserFlo MicroShunt group, and from 21.1 mmHg to 11.1 mmHg, with a mean of 0.3 postoperative medications, in the trabeculectomy group. Fewer postoperative interventions were required for the PreserFlo MicroShunt (40.8% of patients) than for trabeculectomy (67.4%) by year 1. The safety profile was acceptable in both groups, with the incidence of hypotony higher for trabeculectomy at 49.6% compared to 28.9% for the PreserFlo MicroShunt. Vision-threatening complications were similar in both groups, occurring in 1.0% and 0.8% of the PreserFlo MicroShunt and trabeculectomy patients, respectively.

The PreserFlo MicroShunt implantation is a simple technique that does not require scleral dissection, sclerostomy, iridectomy, or tensioning sutures. It is a safe and effective implant for predictable IOP reduction in patients with different glaucoma types. Although it differs from most MIGS, as the PreserFlo MicroShunt requires an ab externo approach, it still fulfills all the other characteristics of this type of surgery with better IOP-lowering effectiveness [30]. Complications are mostly transient, with infrequent postoperative interventions, and compare favorably with traditional filtering surgeries. The MMC dose and placement also play an important role in the surgical results. The PreserFlo MicroShunt, either alone or in combination with phacoemulsification, is a valuable option for patients with open-angle glaucoma at different stages, reducing the number of antiglaucoma medications [52]. Other potential advantages of the PreserFlo Microshunt over traditional glaucoma procedures include faster recovery time, less impact on leisure activities (such as swimming), and reduced risk of damaging other structures in the eye that can necessitate additional ocular surgery. Most MIGS/MIBS are not designed to replace trabeculectomy. However, according to a survey conducted in the United Kingdom [53], the PreserFlo MicroShunt has seen rapid adoption among glaucoma surgeons, with half of the study’s respondents using this device, despite the lack of long-term data on its effectiveness. They predicted that its usage will significantly increase, while the use of trabeculectomies will decrease. Glaucoma consultants report that the implantation of the device is quicker to learn, can be performed faster, and requires fewer postoperative follow-ups and interventions than trabeculectomy, which may facilitate a more efficient service delivery for patients requiring surgery. Traditional bleb surgery is highly effective; however, it carries the risk of postoperative refractive/astigmatic changes, bleb-related postoperative ocular surface changes, rare but potentially sight-threatening complications, impact on the quality of life, and unpredictability of the IOP-lowering effect.

## 5. Conclusions

MIBS with PreserFlo MicroShunt seems to have long-term efficacy (low teens and medication-free), fast postoperative recovery, low impact on refractive error, and low frequencies of postoperative visits and interventions (needling and revision). This approach also ensures a consistent technique and reduced variability. Future studies are needed to determine the exact indications for PreserFlo MicroShunt implantation, including the types of glaucoma, stages of the disease, long-term effects and safety profile, endothelial cell loss, and technical details, such as the time of antifibrotic agent application.

## Figures and Tables

**Table 1:** Characteristics of commercially developed MIGS devices.

Device	Manufacturer	Year of Certification	Size and Structure	Surgical Technique	Mechanism of Action
First-generation iStent^®^	Glaukos, San Clemente, CA, USA	European Union CE certified & FDA approved, 2012	Heparin-coated titanium stent, 1 mm long and 0.33 mm in height, with a “snorkel” and a diameter of 120 µm, “L”-shaped.	Ab interno implantation, using a preloaded injector, placed through the TM into the SC.	Improves AH outflow from the AC into the SC, bypassing the site of highest outflow resistance. It can be implanted alone (solo procedure) or simultaneously with phacoemulsification (combined procedure).
iStent inject W^®^	Glaukos Corporation, Laguna Hills, CA, USA	FDA approved, 2018	The system contains an injector preloaded with two heparin-coated titanium stents, 0.23 × 0.36 mm each, with a central lumen (80 µm in size) and four side outlets (50 µm in size).	The implants are placed ab interno into two distinct areas (with three clock hours between the two insertion sites) of the TM into the SC.	The head of the device has four side outlets in addition to the central one, theoretically allowing multidirectional aqueous outflow, where AH subsequently flows into collector channels. The two stents can tap into up to 6 clock hours of the SC.
iStent Supra^®^	Glaukos Corporation, Laguna Hills, CA, USA	-	Third-generation suprachoroidal stent made of polyethersulfone and titanium, with a heparin-coated lumen of approximately 165 µm in diameter and 4 mm in length.	Is positioned ab interno through the TM into the suprachoroidal space.	Increasing aqueous outflow via the uveoscleral pathway.
Hydrus^®^	Ivantis, Inc, Irvine, CA, USA	European Union CE certified & FDA approved, 2016	The microstent is made of nitinol, a nickel-titanium alloy. It is an approximately 8 mm-long, curved device.	Flexible aqueous drainage device designed to be placed ab interno, where it bypasses the TM and dilates approximately three clock hours of SC. The inlet remains in the AC, while the remainder of the device is placed into the SC.	The Hydrus design serves to provide an alternate AH route that otherwise faces resistance at the juxtacanalicular segment of the TM and SC inner wall, and further provides an intracanalicular scaffold for the SC, providing an outflow route to multiple collector channels.
XEN^®^ Gel Stent	Allergan INC, Dublin, Ireland	CE certified, 2011	A 6 mm tube, made of porcine-gelatin cross-linked with glutaraldehyde. Originally three versions were created: Xen 140, Xen 63, and Xen 45. All three are 6 mm long, but they differ in their lumen diameters: 140 μm, 63 μm, and 45 μm. The commercially available type is XEN 45.	Is implanted via an ab interno approach with an injector. The use of antifibrotic agents is recommended.	It drains AH from the AC to a fornix-based subconjunctival/Tenon’s flap.
CyPass^®^ Micro-Stent	Alcon, Fort Worth, TX, USA	Recalled by the FDA due to concerns raised for endothelial cell loss after 5 years of follow-up	Polyamide implant, 6.35 mm in length and 510 μm in external diameter.	Implanted ab interno into the suprachoroidal space.	The purpose of this stent was to achieve controlled AH outflow from the AC into the suprachoroidal space.
MINIject	iSTAR Medical SA, Wavre, Belgium	European Union CE certified, 2021	The MINIject implant is made of biocompatible STAR material, which is soft and flexible medical-grade silicone that conforms to the anatomic features of the eye.	The device is inserted into the nasal quadrant of the eye through a 2 mm clear corneal incision.	Designed to reduce IOP by enhancing AH outflow from the AC into the supraciliary space.
Goniotomy with Kahook Dual Blade	KDB, New World Medical Inc, Rancho Cucamonga, CA, USA	FDA approved, 2015	The sharp tip is designed with a taper to allow for smooth entry of the blade through the TM and into the SC. The heel of the device fits easily within the SC.	Is introduced into the SC through a temporal clear corneal incision.	After piercing the TM with the tip of the device, it is advanced forward along the length of the TM to enter the SC in the nasal quadrant of the eye. The dual blades create parallel incisions of the TM, allowing the excision of a strip of the TM, which achieves a nearly complete TM removal, to improve AH outflow into the SC.
GATT		Introduced by Grover in 2015	Uses a 5–0 prolene suture or microcatheter.	The TM is cleaved directly behind Schwalbe’s line, creating the so-called trabecular shelf.	The purpose of the GATT is to create an opening in the TM, thereby reducing the proximal resistance to aqueous outflow.
Ab interno canaloplasty	Ellex Medical Lasers Pty Ltd., Adelaide, Australia	Introduced by Gallardo in 2018	Is performed under gonioscopic view with the iTrack™ 250 μm microcatheter.	Is inserted through a clear corneal incision into the AC. The tip of the device is used to penetrate the TM and create an opening to the SC. The iTrack device is advanced through the canal’s whole circumference, and a viscoelastic is injected during its withdrawal.	Improves the conventional AH outflow.
Endoscopic cyclophotocoagulation	Endo Optiks, Little Silver, NJ, USA	Developed by Martin Uram in 1992	Combines a 175 W xenon light source for illumination, 810 nm diode laser for photocoagulation, helium-neon laser aiming beam, and video imaging for intraocular visualization. The endoscopy probe contains all three fiber groupings and is available in 19, 20, or 23 gauge sizes, with a field of view ranging from 70° to 140° and a depth of focus spanning 1–30 mm.	The probe tips are either straight or curved and fit easily through a 2.0 mm clear corneal incision. The advantage of this approach over standard transscleral cyclophotocoagulation and both micropulse and ultrasound cyclophotocoagulation is the precise vision-guided destruction of a certain number of ciliary processes.	A MIGS procedure that uniquely lowers IOP through the reduction of AH production with clear corneal access or through the pars plana of the ciliary body.
OMNI surgical system	Sight Sciences, Inc, California, USA	FDA clearance in December 2017	A handle with curved cannula and beveled tip. It contains the internal reservoir for viscoelastic, which is poured during the retraction of the microcatheter.	The OMNI surgical system facilitates the microcatheterization of the SC circumferentially from a single clear corneal incision, allowing the surgeon to perform ab interno canaloplasty followed by trabeculotomy using a single fully integrated handheld system.	Combining the two different mechanistic modalities successively addresses multiple points of outflow resistance in the conventional outflow pathway, both proximal (i.e., juxtacanalicular and inner wall of the SC) and distal (i.e., the SC and collector channels).

AC, anterior chamber; AH, aqueous humor; FDA, US Food and Drug Administration; GATT, gonioscopy-assisted ab interno trabeculotomy; IOP, intraocular pressure; KDB, Kahook Dual Blade; MIGS, microinvasive glaucoma surgery; SC, Schlemm’s canal; TM, trabecular meshwork.

## Data Availability

All data were placed in the manuscript.

## References

[1] Gedde S.J., Schiffman J.C., Feuer W.J., Herndon L.W., Brandt J.D., Budenz D.L. (2012). Treatment outcomes in the Tube Versus Trabeculectomy (TVT) study after five years of follow-up. Am. J. Ophthalmol..

[2] Gedde S.J., Herndon L.W., Brandt J.D., Budenz D.L., Feuer W.J., Schiffman J.C. (2012). Postoperative complications in the Tube Versus Trabeculectomy (TVT) study during five years of follow-up. Am. J. Ophthalmol..

[3] Soltau J.B., Rothman R.F., Budenz D.L., Greenfield D.S., Feuer W., Liebmann J.M., Ritch R. (2000). Risk factors for glaucoma filtering bleb infections. Arch. Ophthalmol..

[4] SooHoo J.R., Seibold L.K., Radcliffe N.M., Kahook M.Y. (2014). Minimally invasive glaucoma surgery: Current implants and future innovations. Can. J. Ophthalmol..

[5] Jabłońska J., Lewczuk K., Konopińska J., Mariak Z., Rękas M. (2022). Microinvasive glaucoma surgery: A review and classification of implant-dependent procedures and techniques. Acta Ophthalmol..

[6] Saheb H., Ahmed I.I. (2012). Micro-invasive glaucoma surgery: Current perspectives and future directions. Curr. Opin. Ophthalmol..

[7] Fellman R.L., Mattox C., Singh K., Flowers B., Francis B.A., Robin A.L., Butler M.R., Shah M.M., Giaconi J.A., Sheybani A. (2020). American Glaucoma Society Position paper: Microinvasive glaucoma surgery. Ophthalmol. Glaucoma.

[8] Caprioli J., Kim J.H., Friedman D.S., Kiang T., Moster M.R., Parrish R.K., Rorer E.M., Samuelson T., Tarver M.E., Singh K. (2015). Special commentary: Supporting innovation for safe and effective minimally invasive glaucoma surgery: Summary of a joint meeting of the American Glaucoma Society and the Food and Drug Administration, Washington, DC, February 26, 2014. Ophthalmology.

[9] Craven E.R., Katz L.J., Wells J.M., Giamporcaro J.E. (2012). Cataract surgery with trabecular micro-bypass stent implantation in patients with mild-to-moderate open-angle glaucoma and cataract: Two-year follow-up. J. Cataract Refract. Surg..

[10] Arriola-Villalobos P., Martínez-de-la-Casa J.M., Díaz-Valle D., Fernández-Pérez C., García-Sánchez J., García-Feijoó J. (2012). Combined iStent trabecular micro-bypass stent implantation and phacoemulsification for coexistent open-angle glaucoma and cataract: A long-term study. Br. J. Ophthalmol..

[11] Qidwai U., Jones L., Ratnarajan G. (2022). A comparison of iStent combined with phacoemulsification and endocyclophotocoagulation (ICE2) with the PreserFlo MicroShunt and XEN-45 implants. Ther. Adv. Ophthalmol..

[12] Gambini G., Carla M.M., Giannuzzi F., Caporossi T., De Vico U., Savastano A., Baldascino A., Rizzo C., Killian R., Caporossi A. (2022). PreserFlo^®^ MicroShunt: An Overview of This Minimally Invasive Device for Open-Angle Glaucoma. Vision.

[13] Dhingra D., Bhartiya S. (2020). Evaluating glaucoma surgeries in the MIGS context. Rom. J. Ophthalmol..

[14] Shalaby W.S., Lam S.S., Arbabi A., Myers J.S., Moster M.R., Kolomeyer N.N., Razeghinejad R., Shukla A.G., Hussein T.R., Eid T.M. (2021). iStent versus iStent inject implantation combined with phacoemulsification in open angle glaucoma. Indian J. Ophthalmol..

[15] Paletta Guedes R.A., Gravina D.M., Paletta Guedes V.M., Chaoubah A. (2020). iStent Inject (second-generation trabecular microbypass) versus nonpenetrating deep sclerectomy in association with phacoemulsification for the surgical treatment of open-angle glaucoma and cataracts: 1-year results. J. Glaucoma.

[16] Igarashi A., Ishida K., Shoji N., Chu A., Falvey H., Han R., Ueyama M., Onishi Y. (2022). iStent inject^®^ and cataract surgery for mild-to-moderate primary open angle glaucoma in Japan: A cost-utility analysis. Int. J. Ophthalmol..

[17] Myers J.S., Masood I., Hornbeak D.M., Belda J.I., Auffarth G., Jünemann A., Giamporcaro J.E., Martinez-de-la-Casa J.M., Ahmed I.I.K., Voskanyan L. (2018). Prospective evaluation of two iStent^®^ trabecular stents, one iStent Supra^®^ suprachoroidal stent, and postoperative prostaglandin in refractory glaucoma: 4-year outcomes. Adv. Ther..

[18] Samet S., Ong J.A., Ahmed I.I.K. (2019). Hydrus microstent implantation for surgical management of glaucoma: A review of design, efficacy and safety. Eye Vis..

[19] Dorairaj S., Tam M.D. (2019). Kahook Dual blade excisional goniotomy and goniosynechialysis combined with phacoemulsification for angle-closure glaucoma: 6-month results. J. Glaucoma.

[20] Grover D.S., Godfrey D.G., Smith O., Feuer W.J., Montes de Oca I., Fellman R.L. (2014). Gonioscopy-assisted transluminal trabeculotomy, ab interno trabeculotomy: Technique report and preliminary results. Ophthalmology.

[21] Kicińska A.K., Danielewska M.E., Rękas M. (2022). Safety and efficacy of three variants of canaloplasty with phacoemulsification to treat open-angle glaucoma and cataract: 12-month follow-up. J. Clin. Med..

[22] Hughes T., Traynor M. (2020). Clinical results of ab interno canaloplasty in patients with open-angle glaucoma. Clin. Ophthalmol..

[23] Gallardo M.J., Supnet R.A., Ahmed I.I.K. (2018). Viscodilation of Schlemm’s canal for the reduction of IOP via an ab-interno approach. Clin. Ophthalmol..

[24] Wiącek M.P., Miszczuk T., Lipiński A., Machalińska A. (2021). Safety and efficacy of isolated endoscopic cyclophotocoagulation in pseudophakic patients with primary open-angle glaucoma-12-month follow-up. J. Clin. Med..

[25] Seibold L.K., SooHoo J.R., Kahook M.Y. (2015). Endoscopic cyclophotocoagulation. Middle East Afr. J. Ophthalmol..

[26] Vold S.D., Williamson B.K., Hirsch L., Aminlari A.E., Cho A.S., Nelson C., Dickerson J.E. (2021). Canaloplasty and trabeculotomy with the OMNI system in pseudophakic patients with open-angle glaucoma: The ROMEO Study. Ophthalmol. Glaucoma.

[27] Fili S., Seddig S., Vastardis I., Perdikakis G., Wölfelschneider P., Kohlhaas M. (2021). Explantation of the CyPass implant in a case series of patients with corneal decompensation. Ophthalmologe.

[28] Denis P., Hirneiß C., Reddy K.P., Kamarthy A., Calvo E., Hussain Z., Ahmed I.I.K. (2019). A first-in-human study of the efficacy and safety of MINIject in patients with medically uncontrolled open-angle glaucoma (STAR-I). Ophthalmol. Glaucoma.

[29] Denis P., Hirneiß C., Durr G.M., Reddy K.P., Kamarthy A., Calvo E., Hussain Z., Ahmed I.K. (2022). Two-year outcomes of the MINIject drainage system for uncontrolled glaucoma from the STAR-I first-in-human trial. Br. J. Ophthalmol..

[30] Wagner F.M., Schuster A.K., Emmerich J., Chronopoulos P., Hoffmann E.M. (2020). Efficacy and safety of XEN^®^-implantation vs. trabeculectomy: Data of a “real-world” setting. PLoS ONE.

[31] Pinchuk L., Riss I., Batlle J.F., Kato Y.P., Martin J.B., Arrieta E., Palmberg P., Parrish R.K., Weber B.A., Kwon Y. (2017). The development of a micro-shunt made from poly(styrene-block-isobutylene-block-styrene) to treat glaucoma. J. Biomed. Mater. Res. B Appl. Biomater..

[32] Acosta A.C., Espana E.M., Yamamoto H., Davis S., Pinchuk L., Weber B.A., Orozco M., Dubovy S., Fantes F., Parel J.M. (2006). A newly designed glaucoma drainage implant made of poly(styrene-b-isobutylene-b-styrene): Biocompatibility and function in normal rabbit eyes. Arch Ophthalmol..

[33] Lewczuk K., Jabłońska J., Konopińska J., Mariak Z., Rękas M. (2022). Schlemm’s canal: The outflow ‘vessel’. Acta Ophthalmol..

[34] Beckers J.M., Pinchuk L. (2019). Minimally invasive glaucoma surgery with a new ab-externo subconjunctival bypass—Current status and review of literature. Eur. Ophthalmic. Rev..

[35] Arrieta E.A., Aly M., Parrish R., Dubovy S., Pinchuk L., Kato Y., Fantes F., Parel J.M. (2011). Clinicopathologic correlations of poly-(styrene-b-isobutylene-b-styrene) glaucoma drainage devices of different internal diameters in rabbits. Ophthalmic. Surg. Lasers Imaging.

[36] Silber S., Colombo A., Banning A.P., Hauptmann K., Drzewiecki J., Grube E., Dudek D., Baim D.S. (2009). Final 5-year results of the TAXUS II trial: A randomized study to assess the effectiveness of slow- and moderate-release polymer-based paclitaxel-eluting stents for de novo coronary artery lesions. Circulation.

[37] Ormiston J.A., Charles O., Mann T., Hall J.J., McGarry T., Cannon L.A., Webster M.W., Mishkel G.J., Underwood P.L., Dawkins K.D. (2013). Final 5-year results of the TAXUS ATLAS, TAXUS ATLAS Small Vessel, and TAXUS ATLAS Long Lesion clinical trials of the TAXUS Liberté paclitaxel-eluting stent in de-novo coronary artery lesions. Coron. Artery Dis..

[38] Kappetein A.P., Head S.J., Morice M.C., Banning A.P., Serruys P.W., Mohr F.W., Dawkins K.D., Mack M.J. (2013). Treatment of complex coronary artery disease in patients with diabetes: 5-year results comparing outcomes of bypass surgery and percutaneous coronary intervention in the SYNTAX trial. Eur. J. Cardiothorac. Surg..

[39] Batlle J.F., Fantes F., Riss I., Pinchuk L., Alburquerque R., Kato Y.P., Arrieta E., Peralta A.C., Palmberg P., Parrish R.K. (2016). Three-year follow-up of a novel aqueous humor MicroShunt. J. Glaucoma.

[40] Sadruddin O., Pinchuk L., Angeles R., Palmberg P. (2019). Ab externo implantation of the MicroShunt, a poly (styrene-block-isobutylene-block-styrene) surgical device for the treatment of primary open-angle glaucoma: A review. Eye Vis..

[41] Saeed E., Zalewska R., Konopińska J. (2022). Early complications and results of Preserflo MicroShunt in the management of uncontrolled open-angle glaucoma: A case series. Int. J. Environ. Res. Public Health.

[42] Fine I.H., Hoffman R.S., Packer M. (2004). Bimanual bipolar diathermy for recurrent hyphema after anterior segment intraocular surgery. J. Cataract Refract. Surg..

[43] Optimal Use of Minimally Invasive Glaucoma Surgery: Recommendations. https://www.ncbi.nlm.nih.gov/books/NBK543013.

[44] Batlle J.F., Corona A., Albuquerque R. (2021). Long-term results of the PRESERFLO MicroShunt in patients with primary open-angle glaucoma from a single-center nonrandomized study. J. Glaucoma.

[45] Beckers H.J.M., Aptel F., Webers C.A.B., Bluwol E., Martínez-de-la-Casa J.M., García-Feijoó J., Lachkar Y., Méndez-Hernández C.D., Riss I., Shao H. (2022). Safety and effectiveness of the PRESERFLO^®^ MicroShunt in primary open-angle glaucoma: Results from a 2-year multicenter study. Ophthalmol. Glaucoma.

[46] Fea A.M., Laffi G.L., Martini E., Economou M.A., Caselgrandi P., Sacchi M., Au L. (2022). Effectiveness of MicroShunt in patients with primary open-angle and pseudoexfoliative glaucoma: A retrospective European multicenter study. Ophthalmol. Glaucoma.

[47] Scheres L.M.J., Kujovic-Aleksov S., Ramdas W.D., de Crom R.M.P.C., Roelofs L.C.G.R., Berendschot T.T.J.M., Webers C.A.B., Beckers H.J.M. (2021). XEN^®^ Gel Stent compared to PRESERFLO™ MicroShunt implantation for primary open-angle glaucoma: Two-year results. Acta Ophthalol..

[48] Riss I., Batlle J., Pinchuk L., Kato Y.P., Weber B.A., Parel J.M. (2015). One-year results on the safety and efficacy of the InnFocus MicroShunt™ depending on placement and concentration of mitomycin C. J. Fr. Ophtalmol..

[49] Pawiroredjo S.S.M., Bramer W.M., Pawiroredjo N.D., Pals J., Poelman H.J., de Vries V.A., Wolfs R.C.W., Ramdas W.D. (2022). Efficacy of the PRESERFLO MicroShunt and a Meta-Analysis of the Literature. J. Clin. Med..

[50] Pillunat K.R., Herber R., Haase M.A., Jamke M., Jasper C.S., Pillunat L.E. (2022). PRESERFLO™ MicroShunt versus trabeculectomy: First results on efficacy and safety. Acta Ophthalmol..

[51] Baker N.D., Barnebey H.S., Moster M.R., Stiles M.C., Vold S.D., Khatana A.K., Flowers B.E., Grover D.S., Strouthidis N.G., Panarelli J.F. (2021). Ab-Externo MicroShunt versus trabeculectomy in primary open-angle glaucoma: One-year results from a 2-year randomized, multicenter study. Ophthalmology.

[52] Fili S., Kontopoulou K., Vastardis I., Perdikakis G., Bechrakis N., Kohlhaas M. (2022). PreserFlo™ MicroShunt Combined with Phacoemulsification versus PreserFlo™ MicroShunt as a Standalone Procedure in Patients with Medically Resistant Open-Angle Glaucoma. J. Curr. Ophthalmol..

[53] Kuet M.L., Azuara-Blanco A., Barton K., King A.J. (2022). Will the PRESERFLO™ MicroShunt impact the future of trabeculectomy practice? A UK and Éire Glaucoma Society National Survey. Eye.

